# COVID-19 in adult patients with CHD: a matter of anatomy or comorbidities?

**DOI:** 10.1017/S1047951120001638

**Published:** 2020-06-11

**Authors:** Paolo Ferrero, Isabelle Piazza, Matteo Ciuffreda

**Affiliations:** 1 Pediatric Cardiology and Adult Congenital Heart Disease, Cardiovascular Department, Papa Giovanni XIII Hospital, Bergamo, Italy; 2 Internal Medicine, ASST Papa Giovanni XXIII Hospital, Bergamo, Italy; 3 University of studies of Milan, Milan, Italy

**Keywords:** CHD, COVID-19, pneumonia, comorbidity, anatomy

## Abstract

Little is know about COVID-19 outcome in specific populations such as Adult congenital heart disease (ACHD) patients. We report three cases of adult patients with similar underlying disease with completely different clinical severity at the time of COVID-19 infection. The patient with the most severe clinical course was obese and diabetic, suggesting that COVID-19 mortality and morbidity in Adult congenital heart disease patients might be independent of anatomic complexity.

Patients with cardiac conditions are deemed to be more vulnerable to complication from COVID-19.

Cardiovascular system involvement during SARS-CoV 2 infection has been already reported.^1,2^

Indeed, experts believe that ACHD patients may also be at increased risk of complication from COVID-19 infection, depending on the severity of their underlying condition. They may also present late as signs and symptoms such as dyspnoea and chest discomfort may be attributed to the underlying cardiac condition rather than COVID-19 infection.

We describe three cases of COVID-19 infection in patients with similar anatomy and surgical history, which presented with very different clinical severity. All three patients lived in the middle of the red zone of the Italian outbreak and were admitted in the same period.^3^

## Case 1

The first patient was a 30-year-old man, and his body mass index was 18. He was diagnosed at birth with transposition of great arteries, intact ventricular septum, and pulmonary stenosis. He had undergone atrial switch and interposition of a valved conduit between the sub-pulmonary left ventricle and the pulmonary artery. During follow-up, he had undergone superior pathway stenting and pacemaker implantation. He also had significant chronic lung disease with multiple bronchiectases but normal saturation at rest.

In November 2019, he had a new admission to a peripheral hospital for bacterial pneumonia from which he completely recovered. Echocardiography revealed systemic right ventricle mild dysfunction (tricuspid annular plane systolic excursion = 12 mm) and moderate tricuspid regurgitation. There was no evidence of baffle leak.

On the first of March, he attended the accident and emergency department (AED) of an hospital in the Italian COVID-19 epicentre in Lombardy because of palpitations. Amiodarone and betablokers were prescribed. One week later, he started to suffer from back pain without fever or any other symptoms.

A chest CT showed ground glass opacity and interstitial infiltrates involving the right lung.

Blood tests were almost unremarkable, apart from a mild lymphopenia. His C-reactive protein was also raised. Gas analysis showed a pO2 of 74 mmHg.

A nasopharyngeal swab resulted positive for SARS-CoV-2. He remained afebrile and clinically stable over the following days. Neither oxygen supplementation nor ventilatory support was required. No specific medications were prescribed.

The patients was discharged, clinically back to normal after 8 days. A follow-up nasopharyngeal swab after 14 days was negative.

## Case 2

The second patient was a 48-year-old man (body mass index of 30) who was diagnosed at birth with simple transposition of great arteries. He had undergone Mustard repair at 1 month.

In 2014, he was admitted due to heart failure complicated by kidney disease and diabetes, after being lost at follow-up for many years. Echocardiography showed severe dysfunction of the systemic right ventricle with significant tricuspid regurgitation. Catheterisation disclosed increased pulmonary resistances and reduced cardiac index. He was discharged after conventional heart failure therapy optimisation including furosemide, spironolactone, and bisoprolol,

On the 25th of March, he presented with progressive dyspnoea and fever. Symptoms began 7 days earlier, after a flight. In the AED, he was anxious, tachypneic, and diaphoretic. His respiratory rate was 40/min, blood pressure 130/80 mmHg, HR 80 bpm, oxygen saturation with 15 L/min oxygen through mask with reservoir was 95%. He was admitted to the high-dependency ward.

**Figure 1.**
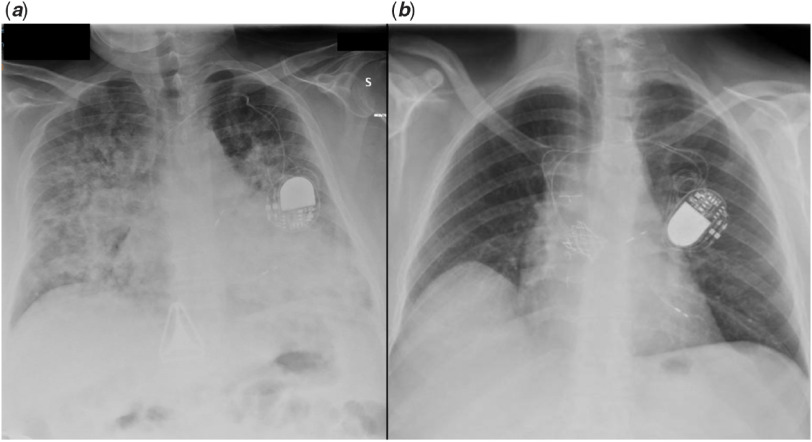
Chest X-ray, frontal view showing diffuse alveolar-interstitial infiltrates in case 2 (***a***) and normal lung fields in case 3 (***b***).

Chest X-ray disclosed bilateral confluent parenchymal infiltrates. (Fig 1a)

Continuous positive airway pressure was started. Arterial gas-analysis with continuous positive airway pressure (FiO2 60% PEEP 10) showed a PO2 oxygen/FiO2 of 200. Table 1 summarises lab results.

**Table 1.** Summary of clinical characteristics and laboratory findings

	Case 1	Case 2	Case 3	
Gender	Male	Male	Male	
Age, y	30	50	48	
BMI kg/m^2^	18	25	33	
Clinical presentation	Atypical	Typical	Typical	
Underline anatomy	TGA/PS	TGA	TGA	
Type of repair	Atrial switch	Atrial switch	Atrial switch	
Diabetes mellitus	No	No	Yes	
Renal failure	No	No	Yes	
Lung disease	Yes	No	No	
Follow-up compliance	Good	Good	Poor	
Flu immunisation	Yes	Yes	No	
Severity of disease	Mild	Moderate	Severe	
Laboratory findings				Normal range
WBC 19^9 g/L	4.2	5.2	6.88	4.2–9.4
Hb g/L	143	122	181	140–170
D-dimer ng/ml	190	13209	324	<500
Creatinin mg/dl	0.61	3.30	0.93	0.3-1.3
GOT U/L	19	592	36	<39
GPT U/L	30	2013	56	<36
CRP mg/dl	57.7	71.2	1.5	<1

BMI = body mass index; CRP = C-reactive protein; GOT = glutamic oxaloacetic transaminase; GPT = glutamic-pyruvic transaminase; TGA = transposition of great arteries; Hb = haemoglobin; WBC = white blood cells

Nasopharyngeal swabs turned out to be positive for SARS-CoV2. High-dose steroids were started achieving progressive clinical improvement that allowed continuous positive airway pressure weaning after 1 week, and chest X-ray also progressively normalised.

## Case 3

The third case was a 48-year-old male (body mass index of 25). The patient had undergone Mustard operation for simple transposition of great arteries. Follow-up was complicated by superior caval pathway obstruction and sinus node dysfunction that required stenting and dual chamber pacemaker implantation. Echocardiographic assessment, performed 6 months before, showed right ventricular moderate dysfunction with mild tricuspid regurgitation.

He presented to the AED on the 6th of April with dry cough and effort dyspnoea. These symptoms had developed 1 week earlier, after few days of low degree fever. His father had been admitted with COVID-19 1 week before the onset of his symptoms. On observation, he was eupneic at rest with normal saturation. Blood pressure was 120/80mmHg and HR 60bpm.

Chest X-ray showed normal transparency of both lungs (Fig 1b). Table 1 reports lab results.

Nasopharyngeal swab turned out to be positive for SARS-CoV2. Due to absence of high-risk criteria, including stable saturation during walking, the patient was discharged after 12 hours of observation. The patients were isolated at home and after 10 days symptoms settled.

## Discussion

COVID-19, in its most typical clinical manifestation, causes interstitial pneumonia and different degree of respiratory failure.^3^

It has been shown that patients with diabetes mellitus and cardiovascular diseases are at increased risk of mortality and complications.^4,5^

However, the epidemiology and clinical course of COVID-19 in patients with CHD is still undefined. The patients here reported, despite a similar anatomy and physiology, had very different clinical course (Table 1).

Experts believe that patients with CHD are at increased risk of complications from COVID-19, although specific determinant of this risk is unknown.^2^ These emblematic cases suggest that underlying anatomic diagnosis per se does not necessarily affect the outcome, but the role of general, already recognised risk factors, such as age, overweight, and extra cardiac comorbidities, might hold relevant for this particular subset of patient.^6^ It is also worth underscoring that ACHD patients may present later and with more severe symptoms presumably because of higher tolerance to dyspnoea and desaturation. On the other hand, in this subset of patients, symptoms might be exacerbated by the underlying cardiac conditions. Interestingly, case 3 was still symptomatic and positive on swab despite almost normal radiological and laboratory findings. Finally, use of specific antiviral agent may portend an additional arrhythmic risk in ACHD patients.

## Conclusions

This study reports three consecutive patients with atrial switch repair for transposition of great arteries and SARS-CoV-2 infection. Although isolated, these experiences reinforce the concept that clinical manifestation may be dramatically different in patients with similar underlying physiology.

## Key Message

Extracardiac comorbidities such as obesity and diabetes mellitus seem to affect the outcome of COVID-19 infection more than specific anatomy in ACHD.
